# Bone marrow necrosis and fat embolism syndrome in sickle cell disease during COVID‐19 infection treated successfully with sequential red cell and plasma exchange

**DOI:** 10.1002/jha2.621

**Published:** 2022-12-15

**Authors:** Syed Rizvi, Muhammad Khakwani, Shivan Pancham, Dimitris Tsitsikas, Zbigniew Rudzki, Ghaniah Hassan‐Smith, Michael Bowen, Christine Wright, Daniel Park

**Affiliations:** ^1^ Department of Acute Internal Medicine University Hospitals Birmingham (UHB) NHS Foundation Trust Birmingham UK; ^2^ Department of Haematology University Hospitals Birmingham (UHB) NHS Foundation Trust Birmingham UK; ^3^ Department of Haematology Sandwell and West Birmingham Hospitals (SWBH) NHS Trust Birmingham UK; ^4^ Department of Haematology Homerton University Hospital (HUH) NHS Foundation Trust London UK; ^5^ Department of Histopathology University Hospitals Birmingham (UHB) NHS Foundation Trust Birmingham UK; ^6^ Department of Neurology University Hospitals Birmingham (UHB) NHS Foundation Trust Birmingham UK; ^7^ Department of Neuro-radiology University Hospitals Birmingham (UHB) NHS Foundation Trust Birmingham UK; ^8^ Department of Haematology Sandwell and West Birmingham Hospitals (SWBH) NHS Trust Birmingham UK; ^9^ Department of Respiratory and Critical Care Medicine University Hospitals Birmingham (UHB) NHS Foundation Trust Birmingham UK

**Keywords:** bone marrow necrosis, COVID19, fat embolism syndrome, neurological impairment, plasma exchange, sickle cell disease

## Abstract

Fat embolism syndrome (FES) is a rare life‐threatening condition that is particularly seen in milder forms of sickle cell disease (SCD). Widespread systemic fat emboli are generated in the context of extensive bone marrow necrosis. Multi‐organ failure with a high morbidity and mortality may quickly develop. Infection with Parvovirus B19 is a common precipitant.

Here, the authors report the case of a 35‐year‐old Afro‐Caribbean man with HbSC disease who presented with FES having tested positive for SARS‐COV‐2. He rapidly became critically ill and required admission to the intensive care unit for organ support. He was treated with red cell exchange and plasma exchange and made a good recovery to leave hospital at week 7.

## 1 CASE PRESENTATION

A 35‐year Afro‐Caribbean man with HbSC disease attended hospital with a short history of generalised body aches, altered mental state and fever. On admission, he had a temperature of 38.4°C, oxygen saturations of 94% on 40% fraction of inspired oxygen (FiO_2_) via venturi mask, a respiratory rate of 35 breaths per minute, tachycardia of 115 beats per minute and a blood pressure of 130/80 mmHg. He rapidly developed progressive type I respiratory failure and reduced conscious level despite an unremarkable computed tomography pulmonary angiogram and a normal computed tomography head. He tested positive for SARS‐COV‐2.

His initial blood tests showed a haemoglobin of 98 g/L, a platelet count 46 × 10^9^/L, a reticulocyte count of 135 × 10^9^/L an elevated white cell count of 21 × 10^9^/L with a predominant neutrophilia, a C‐reactive protein of 178 mg/L and bilirubin 71 μmol/L. A blood film revealed left shifted granulocytes with presence of target cells, infrequent nucleated red blood cells and SC poikilocytes but no evidence of microangiopathic haemolytic anaemia. His haemoglobin S (HbS) was 47.5%, and haemoglobin C (HbC) of 48% performed via high performance liquid chromatography.

**FIGURE 1:**
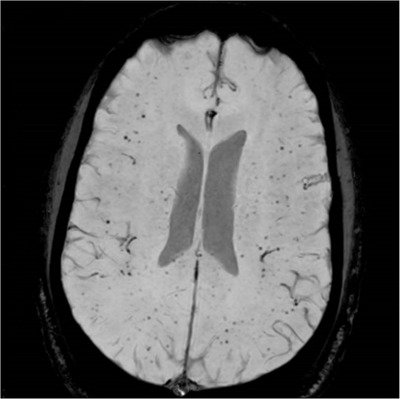
MRI brain with ‘starfield pattern’ microhaemorrhages on axial susceptibility weighted imaging (SWI)

**FIGURE 2:**
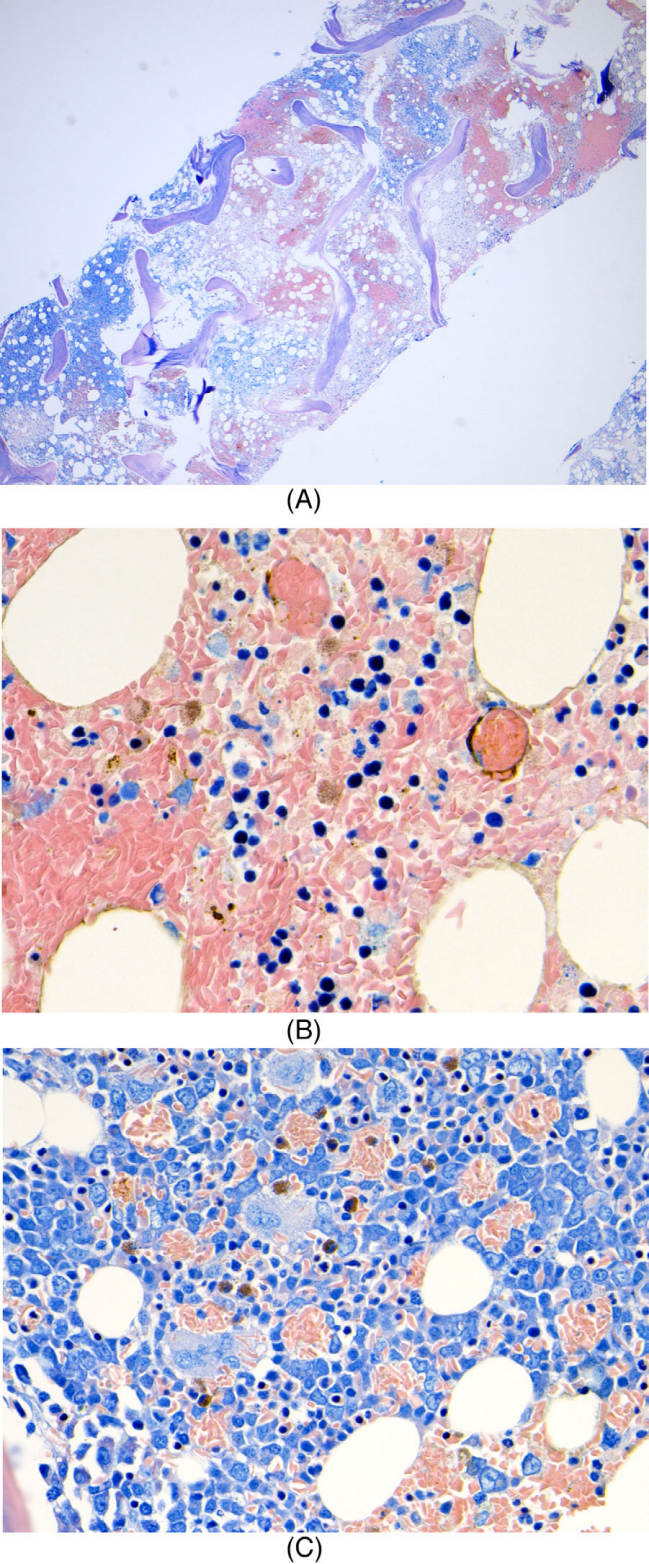
(A)Bone marrow trephine (low power) showing extensive necrosis and haemorrhage; (B) bone marrow trephine (higher power) showing blockage of capillaries and stromal haemorrhage; (C) Giemsa stain showing increased macrophage activity clearing ingested sickle cells

He was admitted to intensive care for non‐invasive ventilation and monitoring and started treatment for presumed meningoencephalitis with ceftriaxone, amoxicillin, aciclovir and dexamethasone. In the following 48 h, his condition deteriorated significantly and developed stage III acute kidney failure, acute liver injury and his Glasgow coma score (GCS) fell to 9. He was electively intubated for airway protection, facilitation of MRI brain and a lumbar puncture. He was also found to be hyperferritinaemic (ferritin 33,500 ng/ml) and developed progressive anaemia with reticulocytopenia (Table 1). He underwent a simple top up transfusion followed by a red cell exchange (RCE), achieving an HbS fraction of 9% post procedure. His point‐of‐care echocardiogram was unremarkable. His cerebrospinal fluid was acellular, whereas his MRI showed multiple areas of restricted diffusion and microhaemorrhages in a so‐called starfield pattern [1, 2] leading to an early suspicion of fat embolism syndrome (FES) (Figure 1). He was discussed in a multi‐disciplinary meeting with hospital intensive care and general physicians, local and regional haematology teams and an external haematologist with expertise in FES in sickle cell disease (SCD). A decision to commence five, one volume plasma exchange (PLEX) sessions with octaplas^TM^, starting on 9th day of hospital admission was reached. Bone marrow histology (Figure 2A–C) later revealed extensive necrosis and haemorrhage, capillaries occluded with sickle cells and areas of increased macrophage activity consistent with the pathophysiology of FES [3, 4].

**TABLE 1:** Haemoglobin g/L (blue line) and reticulocytes x 10^9^/L (orange line)

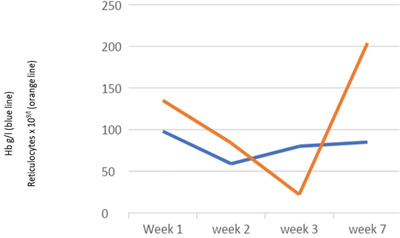

Despite clinical progress being interrupted by resistant hypertension, central venous line infection and clostridium difficile diarrhoea, he continued a slow neurological recovery with normalisation of his full blood count and ferritin (Tables 1 and 2). The patient was weaned with the aid of a tracheostomy. He was decannulated by week 4 and discharged from ICU. At the point of transfer to the ward he was able to sit out in a chair with GCS of 15/15. Modified Medical Research Council grade power was 3−/5 proximal upper limbs, 2+/5 distal upper limbs, 2/5 right lower limb, 2−/5 left lower limb with intact sensations to touch, pain, proprioception and temperature. His neurological disability as measured by the Modified Rankin Scale (MRS) was 4. He was discharged from hospital at week 7 and referred to for further neurorehabilitation. He achieved MRS score of 2 at week 16 follow up.

**TABLE 2:** Ferritin improving trend after treatment with red cell exchange (RCE) and plasma exchange (PLEX)

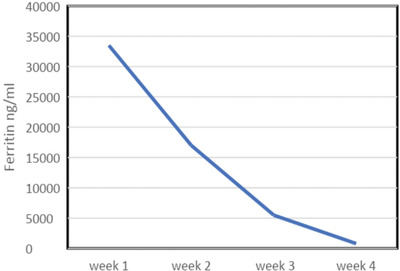

## 2 DISCUSSION

‘Fat embolism’ refers to the presence of fat globules within the lung or peripheral circulation. It is frequently, a consequence of bone fractures and typically has a subclinical course. FES is a more serious complication of fat embolism and seen in about 4% to 10% of cases [5]. The classic FES triad of symptoms comprises type 1 respiratory failure, neurocognitive abnormalities and a petechial rash. FES is more commonly seen after traumatic causes of fat emboli. Non‐traumatic causes mostly involve insult or injury to fatty tissue (including bone marrow necrosis). Severe cases can be fatal. The pathophysiology of FES is not well understood. Emboli may be released into the venous circulation through disrupted venules and venous sinusoids and subsequently reach the systemic circulation through the pulmonary capillaries or a patent foramen ovale. Those such particles have been seen traversing through the inferior vena cava and right heart on echocardiography performed early in the course of the disease would appear supportive of this theory [6]. Alternatively, emboli may be a consequence of free fatty acid aggregation following trauma or illness. Emboli may cause symptoms through mechanical obstruction or by triggering the production of a range of inflammatory mediators [7].

The wide‐ranging clinical manifestations of FES reflect the multi‐organ involvement of this condition. Fat globules in the pulmonary circulation cause tachypnoea, hypoxia and an acute respiratory distress syndrome like picture [8] with nearly half of patients requiring invasive ventilation [9 ]. Neuro‐cognitive abnormalities include mild confusion, headache, convulsions, rigidity, raised intracranial pressure, coma and focal deficits. Dermatological involvement can be transient and may appear very late. A characteristic petechial rash is seen in about one third of patients. This usually involves non‐dependent regions of the body, such as upper trunk, head and neck, axillae and subconjunctivae [9].

Other features of FES include haematological involvement with anaemia, reticulocytopenia and thrombocytopenia seen in upto two thirds of patients [9]. Microangiopathic haemolysis can also occur, and DIC is a rare but important complication of FES that should be considered in patients with unexplained bleeding [10].

FES has been previously described in SCD, although the diagnostic criteria are poorly validated in this group [11, 12]. The precipitating factor is usually thought to be a viral illness, most commonly Parvovirus B19 [13] To our knowledge, this is the first case described in the context of SARS‐CoV‐2 infection.

Genotypes other than HbSS appear to be at greater risk of developing the condition and to have poorer clinical outcomes [13]. An overall mortality in these patients has been reported of up to 64% [13]. Early initiation of treatment with steroids, RCE and possibly PLEX may improve both mortality and neuro‐cognitive impairment [14].

We recommend a high index of suspicion for FES if a patient with a mild sickling disorder presents with unexplained neurological abnormalities, cytopenia, hypoxia or respiratory failure and hyperferritinaemia.

## 3 CONCLUSION

FES is a life‐threatening complication of SCD that can be triggered by COVID‐19 infection. A high index of clinical suspicion is important for timely commencement of treatment. Early RCE and PLEX with comprehensive supportive care and neurorehabilitation are believed to reduce mortality and help prevent severe neurological impairment [15].

## AUTHOR CONTRIBUTIONS

Syed Rizvi coordinated patient care throughout, designed and wrote first manuscript. Muhammad Khakwani provided local haematology specialist input in care of this patient and edited subsequent versions of manuscript, including the preparation of tables. Shivan Pancham, Dimitris Tsitsikas and Christine Wright provided external haematology expert input in care of this patient and critically appraised our manuscript. Zbigniew Rudzki prepared histopathology slides and critically reviewed our manuscript. Ghaniah Hassan‐Smith and Michael Bowen provided expert neurologist and neuroradiology input in patient's care along with critical review of our case study and MRI images. Daniel Park has overseen the whole project and helped revise final version of our manuscript.

## CONFLICTS OF INTEREST

The authors declare they have no conflicts of interest.

## FUNDING INFORMATION

The authors received no specific funding for this work.

## ETHICS STATEMENT

Informed written consent obtained from patient's parent/guardian on their behalf and verbally verified by patient.

## Data Availability

All relevant data for this study included in the manuscript or submitted as supplementary data has been anonymised and is available on reasonable request to the corresponding author SR.
